# Radiosensitization by the Selective Pan-FGFR Inhibitor LY2874455

**DOI:** 10.3390/cells11111727

**Published:** 2022-05-24

**Authors:** Narisa Dewi Maulany Darwis, Eisuke Horigome, Shan Li, Akiko Adachi, Takahiro Oike, Atsushi Shibata, Yuka Hirota, Tatsuya Ohno

**Affiliations:** 1 Department of Radiation Oncology, Gunma University Graduate School of Medicine, 3-39-22, Showa-machi, Maebashi 371-8511, Gunma, Japan; m1920021@gunma-u.ac.jp (N.D.M.D.); ei.horigome@gmail.com (E.H.); m2120014@gunma-u.ac.jp (S.L.); minami0127minaminami@yahoo.co.jp (A.A.); yukahirota@gunma-u.ac.jp (Y.H.); tohno@gunma-u.ac.jp (T.O.); 2 Department of Radiation Oncology, Dr. Cipto Mangunkusumo National General Hospital, Faculty of Medicine Universitas Indonesia, Jl. Diponegoro No. 71, Jakarta Pusat, DKI Jakarta 10430, Indonesia; 3 Gunma University Heavy Ion Medical Center, 3-39-22, Showa-machi, Maebashi 371-8511, Gunma, Japan; 4 Signal Transduction Program, Gunma University Initiative for Advanced Research (GIAR), Gunma University, 3-39-22, Showa-machi, Maebashi 371-8511, Gunma, Japan; shibata.at@gunma-u.ac.jp

**Keywords:** cancer, radiotherapy, radiosensitization, FGFR, LY2874455

## Abstract

Ionizing radiation activates cytoprotective pathways in cancer cells. Fibroblast growth factor receptor (FGFR) is a key player in these pathways. Thus, FGFR signaling is a potential target to induce radiosensitization. LY2874455 is an orally administrable selective pan-FGFR inhibitor. However, the radiosensitizing effects of LY2874455 remain unclear. In this study, we addressed this issue by using radioresistant human cancer cell lines H1703 (*FGFR1* mutant), A549 (*FGFR1–4* wild-type), and H1299 (*FGFR1–4* wild-type). At an X-ray dose corresponding to 50%-clonogenic survival as the endpoint, 100 nM LY2874455 increased the sensitivity of H1703, A549, and H1299 cells by 31%, 62%, and 53%, respectively. The combination of X-rays and LY2874455 led to a marked induction of mitotic catastrophe, a hallmark of radiation-induced cell death. Furthermore, combination treatment suppressed the growth of A549 xenografts to a significantly greater extent than either X-rays or the drug alone without noticeable toxicity. This is the first report to show the radiosensitizing effect of a selective pan-FGFR inhibitor. These data suggest the potential efficacy of LY2874455 as a radiosensitizer, warranting clinical validation.

## 1. Introduction

Photon radiotherapy is one of the most widely used and effective cancer treatments [1]. Recent decades have seen a dramatic advance in radiotherapy technologies toward greater dose conformality, e.g., intensity-modulated radiotherapy and stereotactic body radiotherapy. However, in-field recurrence still occurs due to intrinsic tumor radioresistance, underscoring the need to establish a method of cancer radiosensitization.

Recently, we reported that putative activating mutations in genes encoding fibroblast growth factor receptor (FGFR)1–4 are associated with a worse prognosis for patients with cervical cancer treated with radiotherapy [2]. We also reported that FGFR mutations are enriched in tumors that recur after radiotherapy [3]. Although the data suggest that FGFR-mutated cancers are radioresistant, the prevalence of FGFR mutations among cancers is not high [4]. However, a recent review by Petroni et al. suggests that radiotherapy activates various cytoprotective pathways in cancer cells; therefore, inhibiting such signal transduction pathways is a promising strategy for the radiosensitization of cancers, even those that are genetically wild-type for the corresponding pathways [5].

The human FGFR family comprises five distinct members, FGFR1–4 and FGFR5, encoded by *FGFR1–4* and *FGFRL1*, respectively [6]. FGFR1–4 are receptor tyrosine kinases, whereas FGFR5 lacks the tyrosine kinase domain. FGFRs have various ligands, namely, fibroblast growth factors (FGFs). Canonical FGFs function in an autocrine and paracrine manner upon binding to the extracellular domain of FGFRs. This activates downstream signaling pathways, including mitogen-activated protein kinase (MAPK)-extracellular signal-regulated kinase (ERK), phosphoinositide 3-kinase (PI3K)-protein kinase B (AKT), and Janus kinase (JAK)-signal transducer and activator of transcription (STAT), all of which exert cytoprotective responses to ionizing radiation (IR) [7]. These data indicate the potential of FGFR pathways as a target for radiosensitization. LY2874455 is a selective pan-FGFR inhibitor that can be given orally to humans [8,9,10,11]. However, the radiosensitizing effects of LY2874455 are unknown. Here, we aim to elucidate the radiosensitizing effects of LY2874455 on radioresistant human cancer cells.

## 2. Materials and Methods

### 2.1. Cells and Materials

Human cancer cell lines A549, H1299, and H1703 were obtained from ATCC (Manassas, VA, USA). The genetic status for these cell lines was analyzed using the dataset “Cancer Cell Line Encyclopedia (Broad, 2019)” in cBioPortal [12]. Cells were cultured at 37 °C/5% CO_2_ in RPMI-1640 medium (Sigma-Aldrich, St. Louis, MO, USA) containing 10% fetal bovine serum (Life Technologies, Carlsbad, CA, USA). The original LY2874455 compound was obtained from Cayman Chemicals (Ann Arbor, MI, USA). A stock solution of LY2874455 (20 mM) was prepared by dissolving the original compound in dimethyl sulfoxide (FUJIFILM Wako Chemicals, Osaka, Japan), which was stored at −20 °C. A working solution was freshly prepared from the stock solution prior to each experiment.

### 2.2. Immunoblotting

Immunoblotting was conducted as described previously [13]. Information about the antibodies is summarized in Figure S1. The band intensities were measured using ImageJ (version 1.48, National Institutes of Health, Bethesda, MD, USA) and normalized to those of β-actin (loading control). Uncut immunoblot images are presented in Figure S2.

### 2.3. Irradiation

Cells were irradiated with X-rays using an MX-160Labo (160 kVp, 1.06 Gy/min; mediXtec, Matsudo, Japan) [14]. Mouse tumor xenografts were irradiated with X-rays using a TITAN-225S (200 kVp, 1.30 Gy/min, Shimadzu, Otsu, Japan) [14].

### 2.4. Clonogenic Assays

Clonogenic assays were conducted as described previously [15]. Briefly, cells seeded on 6-well plates were incubated at 37 °C/5% CO_2_ for 12 h. The media were changed to fresh media that contained LY2874455. Cells were incubated for 1 h at 37 °C/5% CO_2_ and then exposed to X-ray irradiation. After incubation at 37 °C/5% CO_2_ for an additional 10 days, cells were fixed with 25% methanol (FUJIFILM), followed by 0.1%-crystal violet staining (Sigma-Aldrich, St. Louis, MO, USA). Colonies of ≥50 cells were recorded using an inverted microscope. The surviving fractions were fitted to the linear–quadratic model, and D_X_ (i.e., the dose that provides X% survival) was calculated [14,16].

### 2.5. DAPI Staining Assays

Radiation-induced clonogenic cell death was evaluated by 4’,6-diamidino-2-phenylindole dihydrochloride (DAPI) staining, as described previously [17,18]. Briefly, cells grown on glass coverslips received the treatment of interest, followed by incubation for 72 h. Cells were then stained with DAPI (Cell Signaling Technology). Using a fluorescence microscope (Eclipse Ni, Nikon, Tokyo, Japan) at ×60 magnification, mitotic catastrophe was determined based on the number of nuclei with two or more distinct lobes; apoptosis was determined based on the presence of apoptotic bodies, nuclear condensation, or fragmentation; and senescence was determined based on the presence of senescence-associated heterochromatic foci. Three hundred cells selected from random fields were evaluated for each experimental condition.

### 2.6. Assessment of Tumor Xenograft Growth

Growth of tumor xenografts was assessed as described previously [14]. Cells (5 × 10^6^ cells) prepared in 100 µL of 0.5% methylcellulose (FUJIFILM) were inoculated subcutaneously into the right thigh of 6-week-old BALB/c female nude mice (Japan SLC, Hamamatsu, Japan). When the tumor volume reached 100 mm^3^, mice were randomized into groups and received oral LY2874455 (3 mg/kg body weight) once daily for 7 consecutive days. One hour after the first drug administration, the xenograft-bearing thighs were irradiated with X-rays (10 Gy) while shielding the rest of the body using lead plates. Tumor size and body weight were measured twice a week. Tumor volume (TV) was calculated using the formula: TV = (L × W^2^)/2, where L and W are the longest diameter and the perpendicular diameter of the tumor, respectively. Measurements were terminated by euthanasia according to standard protocols when a mouse developed severe weakness, metastasis to the skin, or bleeding. All mouse experiments were approved by the Gunma University Animal Experiment Committee (approval number: 18-016; approval date: 5 October 2018).

### 2.7. Statistical Analysis

Differences in cell death among groups were assessed using the Kruskal–Wallis test, followed by a post-hoc pairwise comparison test. Differences in clonogenic survival or tumor growth between groups were assessed by analysis of covariance (ANCOVA), followed by a post-hoc pairwise comparison test [19,20]. A *p*-value <0.05 was considered statistically significant. All statistical analyses were performed using Stata (MP 13, StataCorp, College Station, TX, USA).

## 3. Results

**Figure 1:**
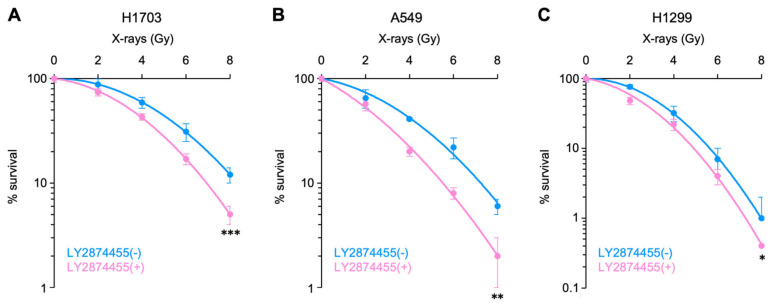
Radiosensitizing effect of LY2874455 in cancer cells. Clonogenic survival of H1703 (**A**), A549 (**B**), or H1299 (**C**) cells exposed to LY2874455 (100 nM) from 1 h pre-irradiation to 10 days post-irradiation is shown (mean ± s.d., *n* = 4). *, *p* < 0.05; **, *p* < 0.01; and ***, *p* < 0.001 (ANCOVA test).

First, we examined the radiosensitizing effects of LY2874455 on radioresistant human cancer cells. For this purpose, we screened in-house radiosensitivity data from 20 cell lines of various origins and selected H1703, A549, and H1299, which showed the lowest sensitivity to photons [14,21,22]. H1703 harbors an amplification of FGFR1 and wild-type FGFR2–4, whereas A549 and H1299 harbor wild-type FGFR1–4 [12]. A previous study reported that LY2874455 induced approximately 50%-clonogenic cell death and approximately 60–70% suppression of ERK phosphorylation, a major downstream signal transducer of FGFR signaling [8], at a dose of approximately 100 nM [3]. Based on these data, we chose to use 100 nM in this study. Notably, LY28774455 sensitized all three cell lines to X-rays in vitro (Figure 1A–C). Analysis of surviving fractions derived from a linear–quadratic model indicated that LY2874455 showed greater cell killing per unit dose at higher doses rather than constant rates of cell killing with increasing dose (Table 1 and Table 2). With D_50_ as the endpoint, LY2874455 increased the sensitivity of H1703, A549, and H1299 cells to X-rays by 31%, 62%, and 53%, respectively (Table 3).

**Figure 2:**
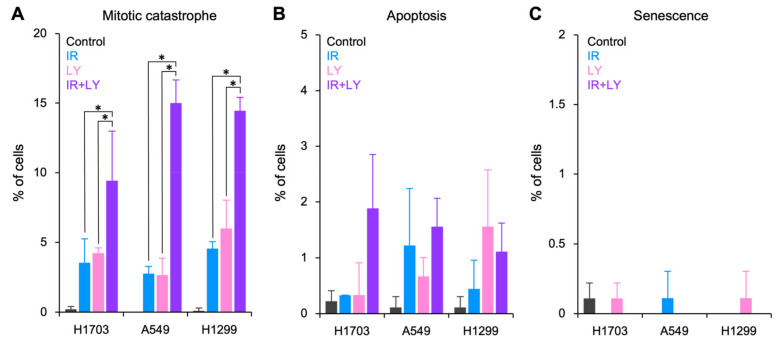
Enhancement of radiation-induced cell death by LY2874455. (**A**) Mitotic catastrophe. (**B**) Apoptosis. (**C**) Senescence. Cells were treated with X-rays (4 Gy) and/or LY2874455 (100 nM) from 1 h pre-irradiation until staining with DAPI 72 h later (mean ± s.d., *n* = 3, 300 cells per experimental setting). IR, X-rays; LY, LY2874455. *, *p* < 0.05 (Kruskal–Wallis test, followed by a post-hoc pairwise comparison test).

**Figure 3:**
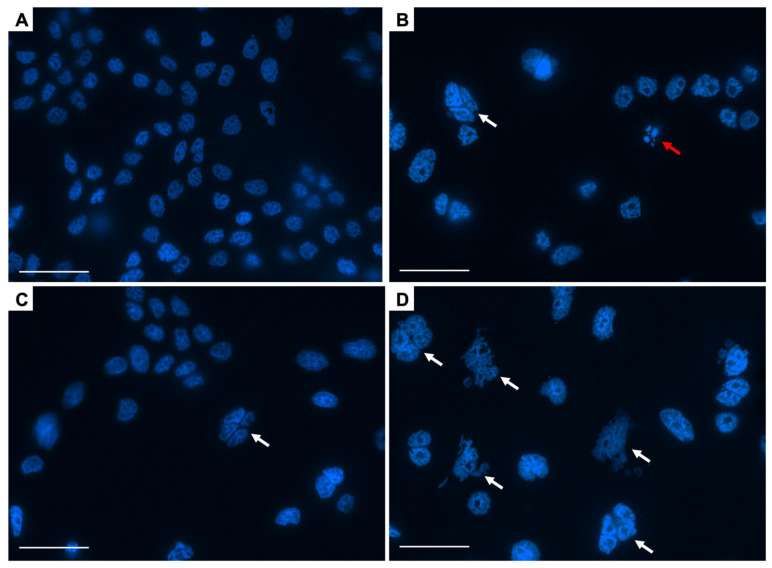
Representative images of DAPI-stained nuclei. A549 cells were treated with X-rays (4 Gy) and/or LY2874455 (100 nM) from 1 h pre-irradiation until staining with DAPI 72 h later. (**A**) No treatment. (**B**) X-rays. (**C**) LY2874455. (**D**) X-rays and LY2874455. Images were obtained using a ×60 lens. White arrows indicate mitotic catastrophe. A red arrow indicates apoptosis. Scale bars, 50 μm.

Next, we conducted a morphological observation of DAPI-stained nuclei to examine the mode of clonogenic cell death induced by combined treatment with X-rays and LY2874455 [18]. Mitotic catastrophe was determined based on the presence of nuclei with two or more distinct lobes [23]. Apoptosis was determined based on the presence of nuclear fragmentation or apoptotic bodies [24]. Senescence was determined based on the presence of senescence-associated heterochromatic foci [25]. Interestingly, combined treatment with X-rays and LY2874455 resulted in a marked induction of mitotic catastrophe, a hallmark of IR-induced cell death (Figure 2A), whereas LY2874455 alone had no effect on the post-IR induction of apoptosis and senescence (Figure 2B,C). Representative images of DAPI-stained nuclei are shown in Figure 3A–D.

**Figure 4:**
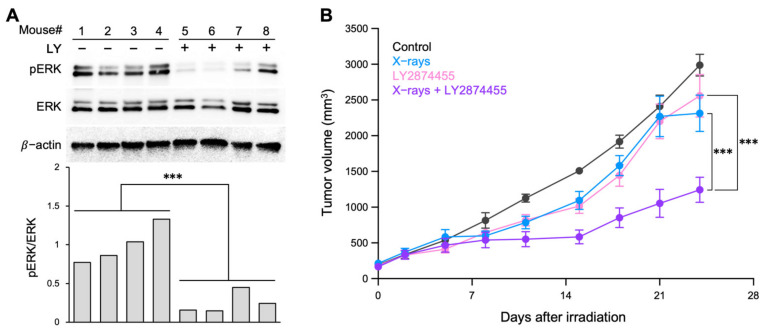
Radiosensitizing effects of LY2874455 in a nude mouse xenograft model. Mice received oral LY2874455 (3 mg/kg body weight) once daily for 7 consecutive days (i.e., Day 1–7). One hour after the first drug administration, tumors were irradiated with X-rays (10 Gy). (**A**) Immunoblots showing suppression of ERK phosphorylation by LY2874455. A549 tumor xenografts were resected from LY2874455-treated or -untreated mice 12 h after the second drug administration (*n* = 4 per group). pERK, phosphorylated ERK. The lower panel shows the quantitation of the immunoblots shown in the upper panel; the ratio of pERK to ERK is shown after normalizing to β-actin. ***, *p* < 0.001 (Mann–Whitney U-test). (**B**) Growth of A549 tumor xenografts (mean ± s.e.m., *n* = 6). ***, *p* < 0.001 (ANCOVA test, followed by a post-hoc pairwise comparison test). IR, X-rays (10 Gy); LY, LY2874455 (3 mg/kg body weight).

Finally, we evaluated the in vivo radiosensitizing effects of LY2874455 in a nude mouse xenograft model using A549 cells (in which the drug showed the highest radiosensitization). Previous studies analyzing the antitumor effects of LY2874455 alone used 3 mg/kg body weight, administered twice daily [8,26,27]. Based on these data and considering the combination treatment with X-rays, we used a milder dose, i.e., 3 mg/kg body weight, administered once daily. At this dose, LY2874455 suppressed ERK phosphorylation, a major downstream signal transducer of FGFR signaling [8], by approximately 75% (Figure 4A). Treatment with X-rays or LY2874455 alone resulted in mild suppression of tumor growth (Figure 4B). By contrast, the combination of X-rays and LY2874455 led to significantly greater suppression of tumor growth than either X-rays or the drug alone (Figure 4B). Importantly, no bodyweight loss or any other toxicity was observed in any treatment group (Figure S3). Taken together, these data suggest that a selective pan-FGFR inhibitor, LY2874455, shows radiosensitizing effects against radioresistant human cancer cells.

## 4. Discussion

Inhibitors of FGFRs are classified as non-selective or selective [6]. Non-selective inhibitors target the ATP-binding cleft of the kinase domains of several growth factor receptors, including FGFR, vascular endothelial growth factor receptor (VEGFR), and platelet-derived growth factor receptor. However, there is general uncertainty about whether these multi-targeting tyrosine kinase inhibitors inhibit FGFRs strongly enough to have clinically relevant effects. In addition, dosing is limited by hypertension, which is caused by inhibition of VEGFRs and by non-specific toxicity [28]. To overcome these issues, selective FGFR inhibitors such as AZD4547, CPL-304-110, debio1347, E7090, infigratinib, pemigatinib, and rogaratinib have been developed [6]. These selective FGFR inhibitors, however, cannot achieve simultaneous inhibition of tyrosine kinases FGFR1–4 because the kinase domain of FGFR4 is structurally distinct from that of FGFR1–3 [29]. LY2874455 was developed as a pan-FGFR inhibitor; it shows a robust inhibition of FGFR4 as well as FGFR1–3 [30,31]; moreover, the drug shows the potent inhibition of gatekeeper FGFR4 mutants [32]. In this study, we found that LY2874455 has a robust radiosensitizing effect on cancer cells. Animal experiments showed no noticeable toxicity upon concomitant use of the drug plus X-rays. To date, few studies have reported the radiosensitizing effects of non-selective FGFR inhibitors [33,34]. Nevertheless, to the best of our knowledge, this is the first study that demonstrates radiosensitization by a selective pan-FGFR inhibitor. Thus, our data highlight FGFR signaling as a promising target for cancer radiosensitization. Since clinical studies of LY2874455 monotherapy show mixed outcomes [9,10,11], the concomitant use of LY2874455 and radiotherapy warrants clinical validation.

The radiosensitizing effects of LY2874455 on FGFR1–4 wild-type cancer cells imply a role for canonical FGF signaling in irradiated cells via the activation of downstream cytoprotective pathways [7]. MAPK-ERK is one of the most crucial signaling pathways that protect cancers against IR-induced death [35,36]. Mitotic catastrophe is a major mode of IR-induced death caused by aberrant mitotic entry by cells harboring unrepaired DNA double-strand breaks (DSBs) [37]. Evidence suggests that ERK is activated in response to IR and increases the expression of RAD51 [36], a key molecule that mediates the homologous-end-joining of DSBs [38]. In line with this, we show here that treatment with LY2874455 enhances IR-induced mitotic catastrophe. Activated ERK also increases the expression of NRF2 [39], a master regulator of cellular antioxidant systems [40]. The induction of DSBs by photons relies predominantly on the so-called indirect effect, which involves the radiolysis of water, followed by the production of reactive oxygen species [14]. From this standpoint, the radiosensitizing effects of LY2874455 may be due, at least partially, to the suppression of NRF2 upregulation, potentially contributing to the enhancement of the indirect effect.

A limitation of this study is that we did not analyze radiosensitization efficacy in association with the detailed mutational status of FGFR1–4, although we used one cell line carrying an FGFR1 amplification (i.e., H1703). Since FGFR1–4 mutant cancers have an unfavorable prognosis [41,42,43], the combination of radiotherapy with FGFR inhibitors may be a viable treatment option for this subset of cancers. A detailed investigation of the radiosensitizing effects of LY2874455 on cells harboring different alterations/mutations in FGFR genes is needed.

## 5. Conclusions

We demonstrate the radiosensitizing effects of LY2874455 against radioresistant human cancer cells both in vitro and in vivo. The data suggest the potential efficacy of LY2874455 as a radiosensitizer, which warrants clinical validation.

## Figures and Tables

**Table 1:** Linear–quadratic model parameters for the survival curves.

Cell Line	LY2874455	α	β	R^2^
H1703	-	−0.0043	0.0335	0.99
	+	0.0582	0.0394	0.99
A549	-	0.0900	0.0315	0.99
	+	0.2643	0.0278	0.99
H1299	-	0.0033	0.0718	0.99
	+	0.1276	0.0696	0.99

R^2^, coefficient of determination.

**Table 2:** Radiosensitizing effect of LY2874455 at each radiation dose calculated based on the linear–quadratic-model-derived surviving fractions.

Dose (Gy)	H1703	A549	H1299
1	6%	15%	11%
2	13%	28%	21%
3	21%	38%	29%
4	29%	47%	37%
5	36%	54%	43%
6	44%	60%	48%
7	51%	64%	53%
8	58%	68%	57%

**Table 3:** Radiosensitizing effect of LY2874455 with D_10_ or D_50_ as the endpoint.

Cell Line		D_10_			D_50_	
	IR alone	IR + LY	Sensitization	IR alone	IR + LY	Sensitization
H1703	8.34	6.93	20%	4.61	3.52	31%
A549	7.24	5.51	31%	3.47	2.14	62%
H1299	5.64	4.97	13%	3.08	2.01	53%

IR, X-rays; LY, LY2874455 (100 nM).

## Data Availability

Data are available from T.O. upon reasonable request.

## Supplementary Materials

The following are available online at https://www.mdpi.com/article/10.3390/cells11111727/s1. Figure S1: Antibodies used for immunoblotting. Figure S2: Uncut images of the immunoblots shown in Figure 4A. Triangles indicate ERK. Figure S3: Body weight of the mice presented in Figure 4B.
