# Supplementary material for: Radiosensitization by the Selective Pan-FGFR Inhibitor LY2874455
Source: Cells. 2022 May 24;11(11):1727. doi: 10.3390/cells11111727 (PMC9179643; doi:10.3390/cells11111727)

Supplementary Figure S1

1st Antibody

Protein	Company	Product ID	Species/type	Dilution rate	Diluent (company)
pERK	Cell Signaling	4370S	rabbit/mAb	1:2000	Can Get Signal Solution 1 (TOYOBO)
ERK	Cell Signaling	9107S	mouse/mAb	1:2000	Can Get Signal Solution 1 (TOYOBO)
b-actin	Cell Signaling	8457T	rabbit/mAb	1:5000	Can Get Signal Solution 1 (TOYOBO)

2nd Antibody

Product	Company	Product ID		Dilution rate	Diluent (company)
Anti-mouse IgG	Cell Signaling	7076S		1:5000	Can Get Signal Solution 2 (TOYOBO)
Anti-rabbit IgG	Cell Signaling	7074S		1:5000	Can Get Signal Solution 2 (TOYOBO)

Supplementary Figure S2

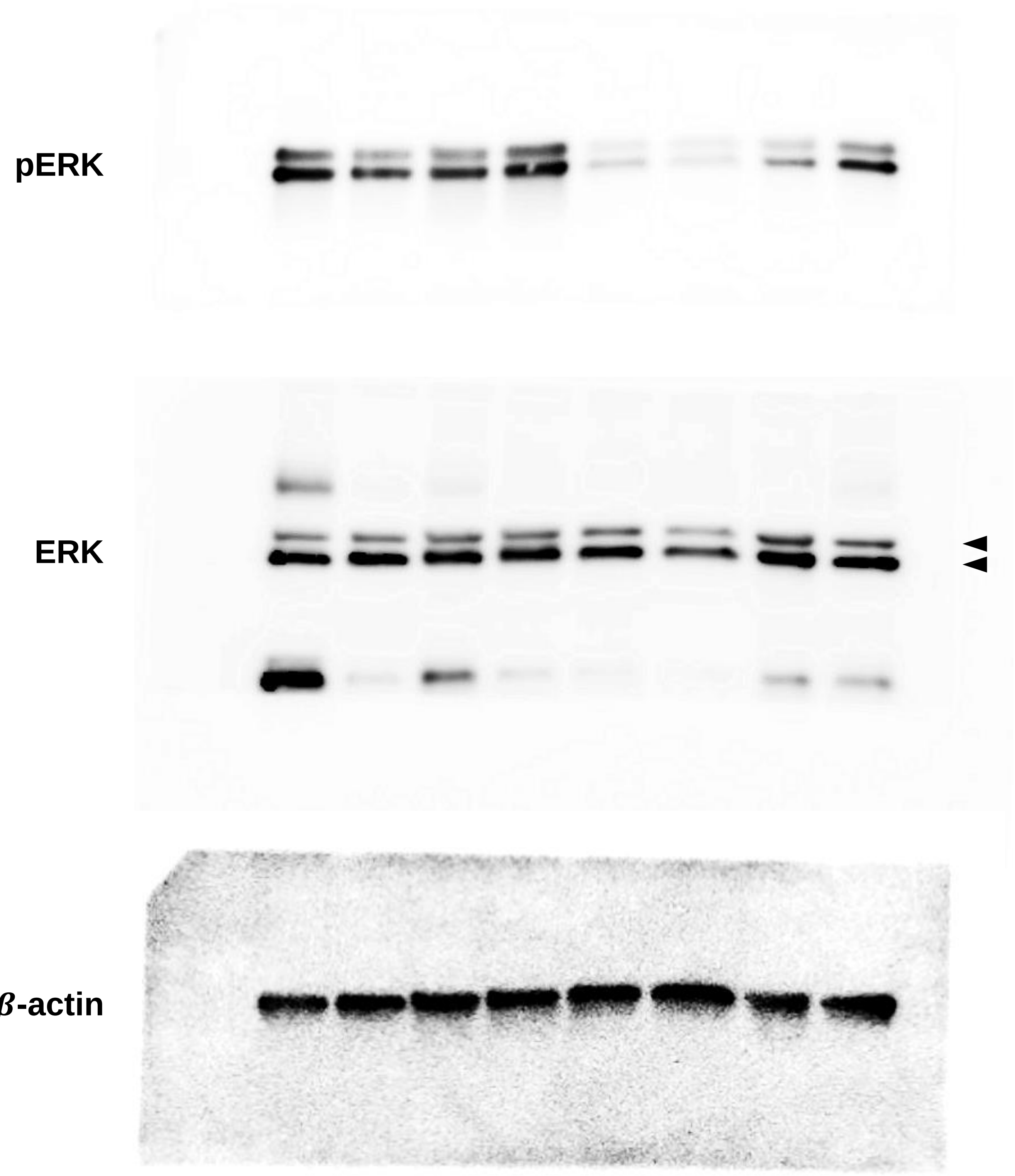

Supplementary Figure S3

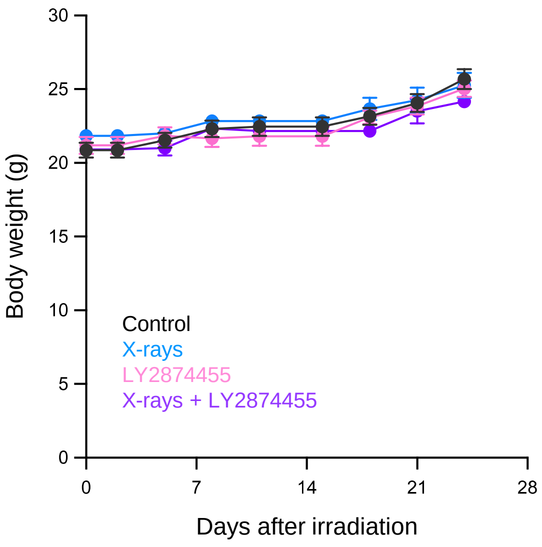

Supplement: Supplementary file 1 [file cells-11-01727-s001.zip › cells-1653055-supplementary.pdf]
